# Maternity Service in Bristol

**Published:** 1952-07

**Authors:** G. G. Lennon


### *Correspondence*

DEAR SIR,

I am already indebted to your *Journal* for the expression of my views on a " Maternity Service in Bristol ". I hope I may be allowed space to answer your correspondents.

The opinions of Dr. Sampson and myself are complementary and not antagonistic, as would appear. He wants " the general practitioner obstetrician as the mainstay of the service, *backed* by easily available, efficient, consultant and emergency services ". I hope he will read my article again and realize that what I have in mind is precisely this, only I used the word " supervised " instead of " backed ". I have already provided for him an emergency obstetric service (" Flying Squad "). I can only hope it is efficient. If I may pursue Dr. Sampson's analogy I would point out that the general practitioner is part of an obstetric team and as in all teams some players get more of the ball than others from the nature of their position on the field!

Dr. Mogg is not clear as to the advantages of a patient coming to a Consultant Clinic twice during her pregnancy although under the care of a general practitioner, yet in my article I pointed out that sometimes indications for admission to hospital had escaped notice. For example, I have seen in Bristol a woman with a major degree of pelvic contraction booked for home confinement. I feel sure that my personal contact with the general practitioners so far must have led them to appreciate my desire for close co-operation with them in this service and keen interest in the place that they should rightly occupy. Dr. Mogg will be interested in the following reasons given by patients as to their desire to have the baby in hospital or at home (Thomas, 1952).

" The reasons given for preferring a hospital confinement are as follows:

| | | |
|---|---|---|
| 1. | Receive better care and attention in hospital and immediate medical help is available when needed | 180 |
| 2. | No relatives to help if at home | 38 |
| 3. | Too nervous to stay at home | 29 |
| 4. | Costs less in hospital | 18 |
| 5. | First baby | 15 |
| 6. | No reason for choosing hospital | 15 |
| 7. | Too much upset to house if she stayed at home | 12 |
| 8. | Get more rest in hospital | 11 |
| | | 318 |

" The reasons given for preferring a home confinement are as follows:

| | | |
|---|---|---|
| 1. | No one available to look after other children while mother is in hospital | 34 |
| 2. | Feel happier at home | 10 |
| 3. | Had previous children at home | 8 |
| 4. | Going into hospital upsets the household too much | 6 |
| 5. | No reason for choice | 24 |
| 6. | Other reasons, e.g. nervous of hospitals; can rest longer at home; feels it is nicer for baby to be born at home, etc." | 10 |
| | | 92 |

It is interesting to see that only eighteen women out of 318 gave as a reason for preferring hospital confinement the fact that it costs less in hospital. It is also interesting that only ten women out of ninety-two felt happier staying at home to have the baby.

It is always dangerous to put one's ideas into writing as one is so often liable to misconstruction. It has been stated that I think all women should be delivered in hospital. This is not my opinion at all. What I am against are the makeshift arrangements for the labour-room in a house. Admittedly in a great majority of cases this arrangement may work well, but where difficulties arise the care of the woman in the home is not easy. I would prefer to see women have their babies in beds attached to hospitals or maternity homes, run by general practitioners and midwives, where proper labour-room facilities exist. When help is required it can then be provided in properly equipped surroundings. In this instance I should like to quote the words of a general practitioner—Dr. A. Talbot Rogers (1952). " If a growing percentage of confinements are admitted to hospital, it should also follow that a growing percentage of the women admitted to hospital will have normal labours, or will have such minor degrees of complication that their delivery will not be outside the competence of the midwife and the general practitioner obstetrician. In fact they will be the very patients that these two obstetric practitioners (separately or as a team) now deliver not unsuccessfully in the patient's own home. The same practitioners could, if allowed, conduct these labours even more efficiently in hospital. Their own competence would be no less for having the added assurance that should more serious complications supervene, specialist obstetric help would be available without delay and without the necessity of transporting a tiring or a gravely shocked patient from home to hospital. . . . The employment of the domiciliary midwife for an appreciable part of her time in the service of the hospital might go a long way towards easing present hospital staffing difficulties. . . . The association of the midwife with the hospital and with the general practitioner obstetrician could also lead to a diminishing need for separate local authority provision of ante-natal care."

It is evident therefore that I would like to see more general practitioner beds available in Bristol—but " supervised ".

G. GORDON LENNON.

### REFERENCES

Thomas, R. C. (1952) Symposium of the Royal Sanitary Institute Health Congress. Margate, 146.

Rogers, A. Talbot (1952), *Ibid* 166.

